# Anthraquinone Emodin Inhibits Tumor Necrosis Factor Alpha-Induced Calcification of Human Aortic Valve Interstitial Cells via the NF-κB Pathway

**DOI:** 10.3389/fphar.2018.01328

**Published:** 2018-11-19

**Authors:** Kang Xu, Tingwen Zhou, Yuming Huang, Qingjia Chi, Jiawei Shi, Peng Zhu, Nianguo Dong

**Affiliations:** ^1^Department of Cardiovascular Surgery, Union Hospital, Tongji Medical College, Huazhong University of Science and Technology, Wuhan, China; ^2^Department of Mechanics and Engineering Structure, Hubei Key Laboratory of Theory and Application of Advanced Materials Mechanics, Wuhan University of Technology, Wuhan, China

**Keywords:** calcific aortic valve disease, anthraquinone, NF-κB, bioinformatic analysis, anti-inflammation

## Abstract

Exploring effective therapies for delaying calcific heart valve disease (CHVD) is the focus of current cardiovascular clinical and scientific research. In this study, human aortic valve interstitial cells (hVICs) were isolated from patients with CHVD. After expansion, cultured hVICs were induced with the tumor necrosis factor-alpha (TNF-α) with or without anthraquinone emodin (EMD) treatments. Cytotoxicity and flow cytometric analysis were used to assess cell growth, while Alizarin Red S staining was used to detect hVICs calcification. Furthermore, RNA-sequencing analysis was utilized to investigate changes in mRNA profiles of cells cultured in TNF-α conditioned medium with or without EMD. Western blotting and qRT-PCR analyses were used for the verification of key selected genes. Our results indicated that EMD had limited influence on hVIC morphology, whereas in a dose-dependent fashion, EMD interfered with hVIC growth under TNF-α conditioned cell culture. Additionally, hVICs treated with TNF-α plus EMD, presented a gradual decrease of positive Alizarin Red S staining, when compared with cells treated with TNF-α only. Notably, cells treated with TNF-α plus EMD showed 1874 differential expression genes (DEGs), among them, 1131 were upregulated and 743 were downregulated. These DEGs displayed an enrichment of biological functions and signaling pathways, among them, *BMP2*, *RELA*, *TNF*, and *TRAF1*, were found to be significantly suppressed by EMD and selected given their role in mediating hVIC calcification. In conclusion, our study shows that EMD inhibits TNF-α-induced calcification and phenotypical transformation of hVICs via the NF-κB signaling pathway, thereby preventing calcification events stimulated during acute inflammatory responses.

## Introduction

Calcific heart valve disease (CHVD) is a common and frequently occurring valvular disease that severely harms human health and is rapidly increasing with the growing aging population (Bonow et al., 2016). Currently, there are no effective preventive measures to treat CHVD, given that the underlying mechanisms of the disease are not very well understood. Statins and ACE inhibitors have been highly hopeful, but many of their large clinical trials have confirmed that neither treatment can effectively slow the progression of this disease (Zhiduleva et al., 2018).

Nevertheless, other studies have shown that early inflammatory responses are considered to be one of the initial critical factors in the occurrence of aortic valvular calcification (Lee et al., 2011). Insights into the cytological basis of CHVD have shown that valve interstitial cells (VICs) are direct participants in the development of valvular calcification (Osman et al., 2006; Meng et al., 2008). In addition, it has been previously reported in the medical and scientific literature, that inflammatory stimuli significantly upregulate the expression of key genes involved in the promotion of valvular calcification in VICs such as *BMP-2*, and *RUNX2*, among others (Nigam and Srivastava, 2009; Yang et al., 2009). For instance, Yu et al. (2011) found that acute inflammatory responses caused by the tumor necrosis factor-alpha, accelerate the calcification of VICs harvested from CAVS patients via the BMP2-Dlx5 pathway. Therefore, the search for effective treatment modalities for valvular calcification, such as the use of systemic drugs to regulate early inflammatory responses, have important clinical value and significance and may effectively delay the onset of heart valvular calcification.

Anthraquinone emodin is a naturally occurring compound, which exhibits beneficial effects for preventing atherosclerosis, due to its anti-inflammatory, anti-proliferatory, and migration properties (Dong et al., 2016). In a recent study, we demonstrated that EMD efficiently inhibited the proliferation of human vascular smooth muscle cells (hVSMCs), when compared to human vascular endothelial cells (hVECs), in a dose-dependent manner (0.05–5 μM) *in vitro*. Similarly, EMD was found to have limited influence on the re-endothelialization of VECs in a rat carotid artery balloon injury model (Xu et al., 2018).

EMD is known to induce cell growth arrest, apoptosis, and autophagy via enhanced ROS production and upregulation of the p53 tumor suppressor protein (Wang et al., 2007). In another study, EMD was shown to inhibit the TNF-α-induced proliferation of human aortic smooth muscle cells (HASMC) via caspase signaling and mitochondrial-dependent apoptotic pathways leading to the downregulation of Bcl-2 and upregulation of Bax protein expression (Heo et al., 2008). Additionally, EMD reduced the TNF-α-induced migration of VSMCs via suppressing the activation of the nuclear factor kappa-light-chain-enhancer of activated B cells (NF-κB) pathway and matrix metalloproteinase-2/9 (MMP-2/9) expression levels (Meng et al., 2012). These findings strongly suggest that EMD has anti-inflammatory effects induced by the actions of TNF-α mediated pathways. At this stage, a large number of studies have focused on the understanding of mechanisms mediating the effects of EMD for the treatment of vascular diseases. Nonetheless, to our knowledge, the effects of EMD in valvular heart disease have not yet been investigated.

Therefore, in this study, we first used TNF-α to induce calcification of human aortic valve interstitial cells. Based on this, we explored whether EMD could effectively inhibit hVICs calcification, and used transcriptome sequencing analysis to explore the signaling mechanisms underlying EMD-mediated activities and gene expression changes induced by TNF-α.

## Materials and Methods

### Cell Culture and Treatments

Human aortic valves were obtained from patients presenting CHVD (according to the calcified classification level 2 standard) (Li et al., 2017), who underwent aortic valve replacement at the Union Hospital, Tongji Medical College, Huazhong University of Science and Technology in Wuhan, China (Table 1). All patients gave written informed consent, and the study was approved by the Ethics Committee of Tongji Medical College, Huazhong University of Science and Technology in Wuhan, China. Human aortic valve specimens were gently cut into small pieces and digested in 2 mg/ml Type I collagenase (Sigma-Aldrich, Saint Louis, MO, United States) for 8 h at 37°C with 5% CO_2_. Subsequently, the undigested tissue was removed using a 70 μm nylon membrane, then the resuspended, separated cells were seeded in high glucose-Dulbecco’s Modified Eagle Medium (DMEM) containing 10% fetal bovine serum (Gibco Laboratories, Gaithersburg, MD, United States) for primary cultures. After expansion, cells from the fourth or fifth passage were used in all the experiments. The treatments used included, 30 ng/ml of TNF-α alone (TNF-α treated group) or TNF-α plus EMD at different concentrations ranging from 1 to 20 μM (TNF-α + EMD treated group). For experiments investigating signaling mechanisms, 10 μM EMD was chosen as the concentration used for RNA-sequencing, qRT-PCR, and western blotting assays.

**Table 1:** Human aortic valve samples.

Sex	Age	Calcific level
F	32	2
F	63	2
M	69	2
M	44	2

*The calcific level was referred to our previous study (Li et al., 2017).*

### Cell Viability and Cytotoxicity Assays

Cell viability and cytotoxicity analyses were performed using the Cell Counting Kit-8 (CCK-8) assay (Sigma-Aldrich). In brief, cells were seeded at a density of 1 × 10^4^ cells/well in 24-well plates. The cells were then treated with 30 ng/ml of TNF-α with or without the different concentrations of EMD (see section above), for 1, 2, and 3 days. After that, at each time point, the cells were washed with phosphate-buffered saline (PBS) and incubated with serum-free medium containing 10% of the CCK-8 reagent. After 2 h of incubation, aliquots were pipetted into 96-well plates and measured at a wavelength of 490 nm using an enzyme labeling instrument (Bio-Rad, Hercules, CA, United States).

### Calcification Inducation Analysis

Cells were seeded into 24-well plates and grown for 3 days until confluency and further cultured in a calcification-inducation culture medium containing 30 ng/ml of TNF-α plus different final concentrations of EMD ranging from 1 to 20 μM for 12 and 18 days, respectively. The degree of cell calcification was measured using Alizarin Red S staining. In brief, cells were incubated in a 100 mM aqueous solution of cetyl-pyridinium chloride and the amount of Alizarin Res S dye released from the extracellular matrix was quantified by spectrophotometry at a wavelength of 550 nm.

### Cell Cycle Flow Cytometric Analysis

Cells were harvested and washed, followed by fixation using 70% alcohol at 4°C overnight. After washing the cells with PBS, the cells were treated with ribonuclease RNase A, then incubated with propidium iodide (PI) for cell cycle analysis on a FACSVerse^TM^ flow cytometer (BD Bioscience, San Jose, CA, United States).

### Detection of mRNA Expression

Cells were harvested using a Trizol reagent (Invitrogen, Carlsbad, CA, United States), followed by RNA isolation. Each sample was reverse transcribed to cDNA according to the protocol (Wang et al., 2018). Then, the reverse transcription product was used as template to perform real-time polymerase chain reaction (PCR) on a StepOne Plus thermal cycler (Applied Biosystems, Foster City, CA, United States) using a PowerUp^TM^ SYBR^TM^ Green Master Mix (Applied Biosystems) following the manufacturer’s guide. All the primers were designed via NCBI primer blast and synthesized by Invitrogen (Table 2). The final data were analyzed by the 2^-ΔΔct^ method.

**Table 2:** List of qPCR primers.

Gene symbol	Accession number	Primer sequence (3′–5′)	Size (bp)
TNF	NM_000594.3	CCTCTCTCTAATCAGCCCTCTG	220
		GAGGACCTGGGAGTAGATGAG	
TRAFl	NM_005658.4	TCCTGTGGAAGATCACCAATGT	117
		GCAGGCACAACTTGTAGCC	
RELA	NM_021975.3	ATGTGGAGATCATTGAGCAGC	151
		CCTGGTCCTGTGTAGCCATT	
RUNX2	NM_001024630.3	CCGCCTCAGTGATTTAGGGC	132
		GGGTCTGTAATCTGACTCTGTCC	
BMP2	NM_001200.3	ACTACCAGAAACGAGTGGGAA	113
		GCATCTGTTCTCGGAAAACCT	
GAPDH	NM_002046.6	ATGCCTCCTGCACCACCAACT	218
		GATGACCTTGCCCACAGCCTTG	

### SDS-PAGE and Western Blotting Assays

After 3 days of treatment with TNF-α (30 ng/ml) with or without EMD (10 μM), the cells were then lysed with standard lysis buffer plus 1% of phenylmethylsulfonyl fluoride (PMSF). Proteins were then separated in 8–12% sodium dodecyl sulfate–polyacrylamide gel electrophoresis (SDS-PAGE) and subsequently transferred to 0.2 μm polyvinylidene fluoride (PVDF) membranes (Bio-Rad). Then the membranes were incubated overnight at 4°C with primary TRAF1 (26845-1-AP) and RELA (10745-1-AP) antibodies (Proteintech, Rosemont, IL, United States). After washes with TBST, the membranes were incubated with the horseradish peroxidase (HRP)-conjugated secondary antibody (1:5000; ZSGB-BIO) for 1 h at 37°C. The targeted proteins were detected in Pierce ECL Western Blotting Substrates (Thermo Scientific, Waltham, MA, United States).

### Detection of mRNA Profiles

RNA-sequencing (RNA-seq) technology was utilized to investigate changes in cell mRNA profiles among the different treatments performed. Isolated RNA was sent to BGI Co., LTD. (Shenzhen, Guangdong, China) for RNA-seq performed on BGISEQ-500, and all the samples were replicated three times for confirmation purposes. Sequencing results were further analyzed using the “R Project” in order to identify DEGs, and perform GO and KEGG pathway enrichment analysis.

### Statistical Analysis

Sequencing results were further analyzed using the R Project, and all other data were analyzed using IBM’s Statistical Package for Social Sciences (SPSS), expressed as the mean ± standard deviation (SD). Statistical comparisons were made by analysis of variance (ANOVA) to check differences among groups. A *p*-value < 0.05 was accepted as statistically significant.

## Results

### Emodin Regulates hVIC Growth Under TNF-α Conditioned Cultures

**FIGURE 1:**
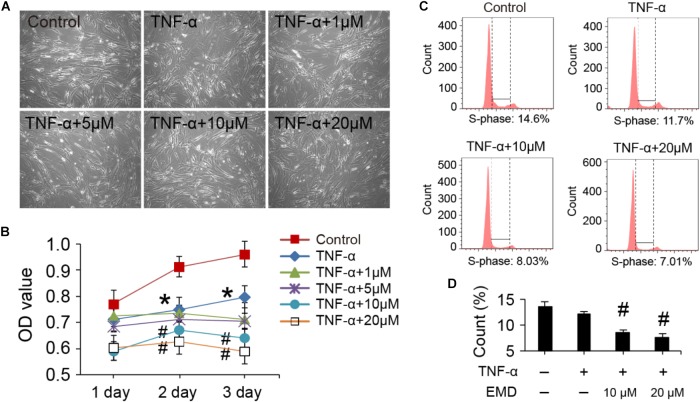
Viability and cell cycle of hVICs with treatments at the concentration of EMD ranging from 1 to 20 μM. **(A)** Morphologies of hVICs with different treatments for 3 days. **(B)** Cell proliferation curve from cck8 assay, *n* = 5. **(C,D)** Cell cycle analysis, (S-phase) were counted and made statistical comparison. ^∗^*p* < 0.05 (vs. control) and #*p* < 0.05 (vs. TNF-α) were accepted as significant difference, *n* = 3.

The viability and cell cycle of hVICs with treatments at the concentration of EMD ranging from 1 to 20 μM. Cell morphologies were monitored with the different treatments for 3 days, and there were no significant changes observed in the basic morphology of the cells, when comparisons between the groups were made (Figure 1A). Furthermore, by analyzing the cell proliferation curve obtained with the CCK-8 assay, TNF-α alone treatments did not appear to significantly cause cell growth arrest after 1 day of treatment. In contrast, cell growth was inhibited when cells were incubated in the presence of TNF-α together with 10 or 20 μM of EMD. Notably, at day two, TNF-α activity began to significantly stagnate cell proliferation compared to the control group (^∗^*p* < 0.05), and the addition of 10 or 20 μM of EMD further inhibited cell proliferation, showing a statistically significant difference when compared to the TNF-α group (#*p* < 0.05). Similarly, at day three this inhibition appears more obvious, as seen in Figure 1B.

Flow cytometry was used to analyze the cell cycle under treatment conditions and demonstrated that cells treated with TNF-α showed suppressed DNA synthesis, with the effect being more detectable after the addition of EMD (Figure 1C). Moreover, statistical comparisons between the two groups showed that supplementing the TNF-α conditioned medium with 10 and 20 μM of EMD significantly affected the S-phase of the cell cycle, when compared with TNF-α treated cells without the addition of EMD (#*p* < 0.05) (Figure 1D). In summary, the addition of EMD to the TNF-α conditioned medium did not appear to cause severe cytotoxicity to hVICs, instead was found to interfere with cell proliferation in a dose dependent manner.

### Emodin Inhibits TNF-α-Induced Calcification of hVICs

**FIGURE 2:**
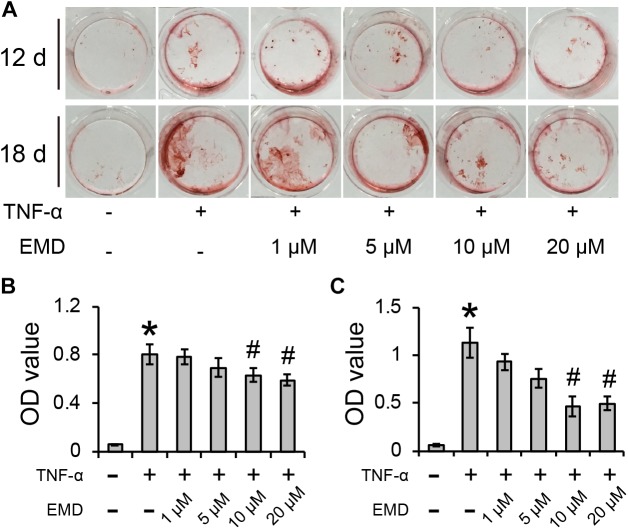
Emodin inhibits TNF-α-induced calcification of hVICs for 12 and 18 days (d). **(A)** Alizarin Red S positive staining. **(B**,**C)** Absorbance value (OD) for 12 days **(B)** and 18 days **(C)** conditioned culuring, ^∗^ and ^#^*p* < 0.05 were accepted as significant difference when, respectively compared to control (absence of both TNF-α and EMD), and to TNF-α group, *n* = 3.

After culturing hVICs with 30 ng/ml of TNF-α for 12 and 18 days, the cells were positively stained with Alizarin Red S and showed significant difference when compared to control cells (^∗^*p* < 0.05) (Figure 2A). The addition of EMD treatments at concentrations ranging from 1 to 20 μM to the TNF-α conditioned medium, resulted in the Alizarin Red S positive staining gradually decreasing in a dose dependent manner. After 12 days of culture in cell conditioned media, although EMD concentrations of 1 and 5 μM did not significantly reduce the calcification deposits observed in the cells, spectroscopy analysis used to measure the Alizarin Red S absorbance of cells treated with 10 and 20 μM of EMD, showed a statistically significant difference when compared with the TNF-α alone group (#*p* < 0.05) (Figure 2B). Similarly, after 18 days of culture in the same cell conditioned media, the trend was more pronounced, and the absorbance of Alizarin Red S in the cells treated with 10 and 20 μM of EMD was significantly reduced by 0.58- and 0.56-fold, respectively, when compared to the TNF-α alone group (#*p* < 0.05) (Figure 2C). Therefore, an EMD concentration of 10 μM was used for further experiments.

### Identification of Differential Expression Genes and Gene Ontology Enrichment Analysis

**FIGURE 3:**
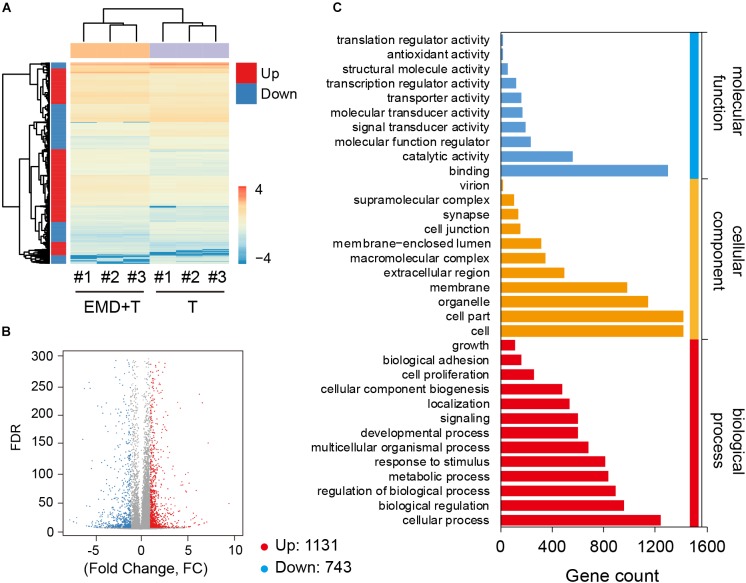
Global gene expression profiles under the TNF-α conditioned culture with or without EMD. **(A)** Heatmap for global gene expression, # indicates number, experiment repeated three times, *n* = 3; **(B)** Volcano map of TNF-α and TNF-α plus EMD expression genes, FC (fold change) > 1 was accepted as positive differentially expressed genes, up for 1131; down for 743. **(C)** GO enrichment of those selected DEGs including biological process, cellular component, molecular function.

Global gene expression profiles exhibited under TNF-α conditioned cultures, demonstrated that with the addition of EMD, great differences of gene expression regulation were observed (Figure 3A), when compared to the TNF-α only group. hVICs from the TNF-α plus EMD conditioned media group showed 1874 DEGs, among them, 1131 DEGs were upregulated and 743 were downregulated (Figure 3B). Furthermore, GO functional annotations were made on the identified DEGs, as seen in Figure 3C. Importantly, molecular function analysis indicated that some of the above DEGs were highly involved in molecular cell adhesion, catalytic activity, and molecular/biological regulatory functions, and enriched in cellular components, including the extracellular region and cell membrane, among others.

### Kyoto Encyclopedia of Genes and Genomes Pathway Enrichment and Analysis

**FIGURE 4:**
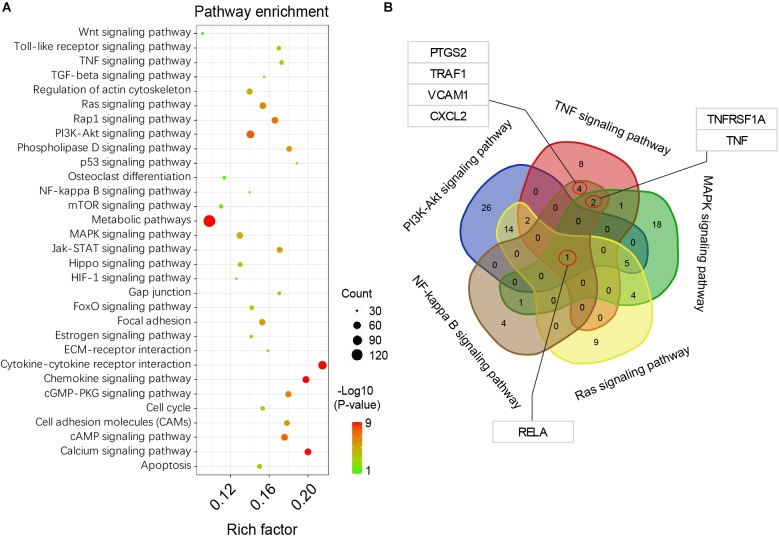
KEGG pathway enrichment analysis. **(A)** pathway enrichment bubble map, a larger *P*-value (-Log10) indicates a higher degree of enrichment. **(B)** Venn interaction of DEGs in the TNF, PI3K-Akt, MAPK, NF-kappa B, and Ras signaling pathways. RELA is the consensus differentially expressed gene.

Based on the DEGs analysis and GO functional annotations described above, KEGG signal pathway enrichment analysis was performed on the identified DEGs described above. Our results showed that these DEGs were highly enriched in functions related to cytokine-cytokine receptor interaction, chemokine and calcium signaling pathways, among others (Figure 4A). Among the DEGs identified and based on TNF, PI3K-Akt, MAPK, NF-κB, and Ras signaling pathways, a consensus of DEGs were selected (Figure 4B). These results showed that the *RELA* gene, is a full-contained consensus DEGs involved in each of the above signaling pathways. In addition, some important functional genes were selected, such as *TNF*, *TNFRSF1A*, *PTGS2*, *TRAF1*, *VCAM1*, and *CXCL2*, among others.

### Selected Genes Expression Profiles and Involving in NF-κB Pathway

**FIGURE 5:**
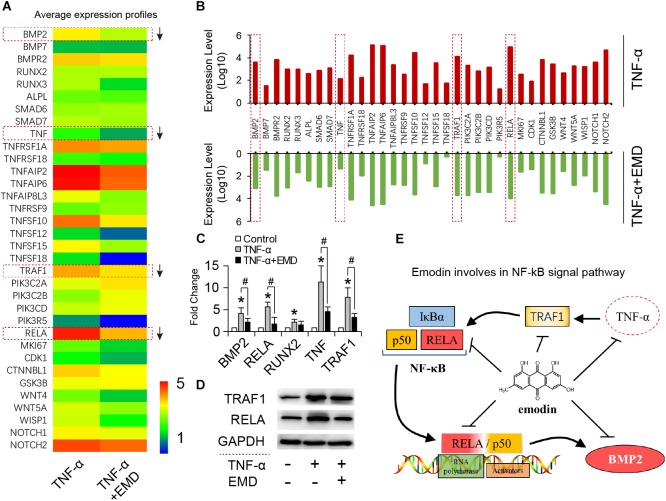
Emodin involves in NF-κB signal pathway. **(A)** Heatmap for 34 typical functional DEGs; **(B)** up-down comparison of the selected gene expression (expression level represents average value, *n* = 3); **(C)** qRT-PCR for detecting BMP2, RELA, RUNX2, TNF, TRAF1 gene expression of hVIC with conditioned culturing, ^∗^ and #*p* < 0.05 were accepted as significant difference when, respectively compared to control (absence of both TNF-α and EMD), and to TNF-α group, *n* = 3; **(D)** Western blotting bands for TRAF1 and RELA protein synthesis, (TNF-α: 30 ng/ml, EMD: 10 μM). **(E)** Graphical indication of emodin involving in NF-κB pathway.

Based on the global gene expression profiles identified, 34 typical functional genes were selected for further investigation. According to the average expression heat map generated (Figure 5A), most of the selected genes in the TNF-α plus EMD group were downregulated compared with the TNF-α only group (Figures 5A,B), among them, the *BMP2*, *RELA*, *TNF*, and *TRAF1* genes were highly inhibited by EMD. Then, qRT-PCR results showed that TNF-α significantly upregulated the expression of these genes, when compared with the blank control group (^∗^*p* < 0.05), and promoted expression genes: *BMP2*, *RELA*, *TNF*, and *TRAF1* were downregulated with presence of EMD (Figure 5C), showed a statistically significant difference (#*p* < 0.05). Furthermore, the protein synthesis of TRAF1 and the key regulatory factor RELA were investigated. These results were consistent with the trend observed with the gene expression profiles described above. As predicted, EMD was able to effectively inhibit the upregulated expression of the TRAF1 and RELA proteins in the presence of TNF-α (Figure 5D). These results indicate that EMD suppresses BMP2 protein expression via the NF-κB pathway by inhibiting TNF, TRAF1, and RELA protein synthesis (Figure 5E).

## Discussion

In a previous study, we found that the EMD compound has the ability to inhibit VSMC proliferation and thereby inhibit intimal hyperplasia (Xu et al., 2018). Since hVICs are similar to VSMCs, both displaying fibroblast-like characteristics, we hypothesize that EMD may be a safe and effective drug for the prevention of cardiovascular diseases.

hVICs grown in TNF-α-induced conditions have a tendency to transform into an osteogenic phenotype, which leads to a significant increase of calcification deposits, accompanied by an upregulation of the *bone morphogenetic protein* (*BMP*)-*2* pro-calcification gene. This is consistent with results reported in the literature (Song et al., 2015; Wang et al., 2015). For instance, Yu et al. (2011) proved that acute inflammatory responses caused by TNF-α activity accelerates the calcification of VICs by up-regulating *BMP2*, *RUNX2*, and other genes mediating osteoblastic differentiation. Our results show that EMD has a significant inhibitory effect on the transformation to a calcification phenotype by hVICs. In order to further understand the mechanism underlying EMD mediated inhibition on calcification, we used RNA-sequencing to analyze the global gene expression of hVICs treated with EMD in the presence of TNF-α induction. In this study, we identified 1874 DEGs with 1131 upregulated genes and 743 downregulated genes. Among them, *TNF*, *TRAF1*, and *RELA* were confirmed to have important roles in the process of calcification inhibition via EMD mediated mechanisms.

TRAF1 is a receptor for TNF, responsible for transducing the signaling pathway across the cell membrane (Tang et al., 2018). Moreover, it has been reported that inflammatory responses can be attenuated by regulating the expression of TRAF1 (McHugh, 2017; Yu et al., 2018). Therefore, it can be hypothesized that inhibition of TRAF1 can effectively slow down the production of inflammatory responses, thereby inhibiting the transcription of downstream genes and the transformation of cellular functions. Markedly, the role of EMD in inhibiting TRAF1 has an excellent regulatory effect on acute inflammatory responses in valvular lesions.

A more important finding is that the expression of RELA at both the gene and protein levels, was significantly inhibited by EMD. RELA, also known as p65, is a REL-associated protein involved in the heterodimer formation, nuclear translocation, and activation of the NF-κB protein (Lee and Burckart, 1998). In our Venn analysis of the different signaling pathways involved, RELA was found to be an intermediate regulator of these pathways, therefore RELA can be considered as a central regulatory factor involved in the calcification inhibition of hVICs mediated by EMD.

## Conclusion

EMD was found to interfere with cell proliferation pathways, while suppressing the calcific transformation of hIVCs under the induction of TNF-α, possibly via the NF-κB signaling pathway by inhibiting the gene expression of *BMP2*, *TNF*, *TRAF1*, and *RELA*.

## Author Contributions

KX, PZ, and QC designed the project, collected the data, and wrote the manuscript. KX, TZ, YH, and JS analyzed the data, wrote and revised the manuscript. ND designed the project, gave financial support, wrote and revised the manuscript. All authors read and approved the final manuscript.

## Conflict of Interest Statement

The authors declare that the research was conducted in the absence of any commercial or financial relationships that could be construed as a potential conflict of interest.

## Abbreviations

ACE: angiotensin-converting enzyme
CAVS: calcific aortic valve stenosis
CHVD: calcific heart valve disease
DEGs: differential expression genes
EMD: emodin
GO: gene ontology
hVICs: human aortic valve interstitial cells
KEGG: kyoto encyclopedia of genes and genomes
MMP: matrix metalloproteinase
ROS: reactive oxygen species
TNF-α: tumor necrosis factor-alpha
VSMC: vascular smooth muscle cell
